# Inhibition of REV‐ERBs stimulates microglial amyloid‐beta clearance and reduces amyloid plaque deposition in the 5XFAD mouse model of Alzheimer’s disease

**DOI:** 10.1111/acel.13078

**Published:** 2019-12-04

**Authors:** Jiyeon Lee, Do Eon Kim, Percy Griffin, Patrick W. Sheehan, Dong‐Hou Kim, Erik S Musiek, Seung‐Yong Yoon

**Affiliations:** ^1^ Department of Brain Science Asan Medical Center University of Ulsan College of Medicine Seoul Korea; ^2^ Department of Neurology Hope Center for Neurological Disorders Washington University School of Medicine St. Louis MO USA

**Keywords:** Alzheimer's disease, circadian, microglia, REV‐ERBs, SR8278

## Abstract

A promising new therapeutic target for the treatment of Alzheimer's disease (AD) is the circadian system. Although patients with AD are known to have abnormal circadian rhythms and suffer sleep disturbances, the role of the molecular clock in regulating amyloid‐beta (Aβ) pathology is still poorly understood. Here, we explored how the circadian repressors REV‐ERBα and β affected Aβ clearance in mouse microglia. We discovered that, at Circadian time 4 (CT4), microglia expressed higher levels of the master clock protein BMAL1 and more rapidly phagocytosed fibrillary Aβ_1‐42_ (fAβ_1‐42_) than at CT12. BMAL1 directly drives transcription of REV‐ERB proteins, which are implicated in microglial activation. Interestingly, pharmacological inhibition of REV‐ERBs with the small molecule antagonist SR8278 or genetic knockdown of REV‐ERBs‐accelerated microglial uptake of fAβ_1‐42_ and increased transcription of BMAL1. SR8278 also promoted microglia polarization toward a phagocytic M2‐like phenotype with increased P2Y_12_ receptor expression. Finally, constitutive deletion of Rev‐erbα in the 5XFAD model of AD decreased amyloid plaque number and size and prevented plaque‐associated increases in disease‐associated microglia markers including TREM2, CD45, and Clec7a. Altogether, our work suggests a novel strategy for controlling Aβ clearance and neuroinflammation by targeting REV‐ERBs and provides new insights into the role of REV‐ERBs in AD.

## Graphical abstract

Genetic and pharmacological inhibition of REV‐ERBα is markedly increased microglial phagocytosis of Aβ by modulating P2Y12R expression with induction of Aβ internalization‐related receptors, leading to the M2 polarization in vitro and in vivo.

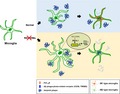

## 1 INTRODUCTION

Circadian rhythms such as the sleep–wake cycle are internal rhythms that exist on a 24‐hr period. These rhythms are generated by the suprachiasmatic nuclei (SCN) in the hypothalamus to integrate environmental cues and modulate diverse biological processes (Mohawk & Takahashi, 2011). Importantly, these rhythms can be disrupted by aging or environmental/genetic changes, leading to abnormal sleep patterns and other physiological and transcriptional disturbances. Circadian rhythm disruption is a symptom of numerous neurological and psychiatric diseases, including Alzheimer's disease (AD) (Coogan et al., 2013; Musiek & Holtzman, 2016). AD is a well‐known neurodegenerative disorder that is accompanied by the accumulation of amyloid‐beta (Aβ) plaques and neurofibrillary tangles in the brain, cognitive impairment, and memory loss (Eriksen & Janus, 2007). The molecular changes associated with AD can be exacerbated by circadian irregularities (Musiek, Xiong, & Holtzman, 2015; Saeed & Abbott, 2017). Indeed, recent studies have revealed that circadian rhythms directly affect Aβ dynamics and pathology (Kress et al., 2018; Schmitt, Grimm, & Eckert, 2017). Despite evidence for the role of the circadian system in Aβ metabolism, the underlying molecular mechanisms for its involvement in AD remain largely unknown.

Components of the cellular circadian clock system are expressed in virtually all cells in the body. The core components of this system, Bmal1 and Clock, heterodimerize and bind to specific *cis*‐regulatory enhancer sequences known as the E‐boxes. These proteins drive the transcription of several clock‐related genes including Period (*Per1/2/3*), Cryptochrome (*Cry1/2*), REV‐ERB proteins (*Nr1d1*/*Nr1d2* encode REV‐ERBα/REV‐ERBβ), and retinoic acid receptor‐related orphan receptors (e.g., *Rora*)*.* Of these, REV‐ERBα and β transcriptionally repress Bmal1 by binding to the RORE *cis*‐element in its promoter region (Herzog, Hermanstyne, Smyllie, & Hastings, 2017) and connect the circadian system to macrophage‐driven inflammation (Gibbs et al., 2012; Griffin et al., 2019; Pariollaud et al., 2018). Rev‐Erbα/β are also nuclear receptors which function as transcriptional repressors and exert a variety of biological functions (Everett & Lazar, 2014; Lam et al., 2013; Woldt et al., 2013). Recent studies suggest that modulating REV‐ERBα activity can be a potent therapeutic target for neurodegenerative disease such as AD via modulating the glia activity and neuroinflammation response (Griffin et al., 2019; Roby et al., 2019).

The initial responders to Aβ accumulation in the brain are innate immune cells known as microglia. Microglia rhythmically express circadian clock genes that can regulate function, including phagocytosis, inflammatory responses, and autophagy (Fonken et al., 2015; Ma, Li, Molusky, & Lin, 2012). This regulation may occur in part by mediating pro‐inflammatory chemokine expression (Lam et al., 2013; Sato et al., 2014). Microglia are highly sensitive to environmental cues and can immediately transform their morphology into distinctive phenotypes, including resting, classically activated (M1), and alternatively activated (M2) microglia (Ma, Wang, Wang, & Yang, 2017; Zhou et al., 2017). M1 polarized microglia are generally associated with pro‐inflammatory cytokine production, while M2 polarization is associated with phagocytosis and neural repair (Cherry, Olschowka, & O'Banion, 2014; Hu et al., 2015).

Microglial activation is also mediated by several purinoceptors (Koizumi, Ohsawa, Inoue, & Kohsaka, 2013). Recently, the purinergic receptor P2Y_12_R, a G_i/o_‐coupled ATP receptor, was proposed as a specific marker for rodent microglia, particularly for the M2 phenotype (Butovsky et al., 2014; Moore et al., 2015; Zhu et al., 2017). Moreover, P2Y_12_R is considered to be a primary receptor that acutely induces microglial chemotaxis toward injury sites or Aβ plaques (Thériault, ElAli, & Rivest, 2015). P2Y_12_R is also implicated in synaptic pruning via modulating microglial phagocytosis. Recent work shows that sleep deprivation disrupted the process of synapse elimination and complement signaling with reduced expression of P2Y_12_R in adolescent but not in adult (Tuan & Lee., 2019). Interestingly, transcription of P2Y_12_R in microglia depends on Bmal1 and circadian‐driven expression of P2Y_12_R controls diurnal morphological changes in cortical microglia (Hayashi, 2013).

We hypothesized that dysregulated clock machinery in microglia might influence microglial behavior in the context of Aβ clearance. In this study, we show a relationship between microglial circadian clock oscillation and Aβ uptake, elucidate the effects of circadian repressors REV‐ERBα/β on Aβ clearance via increased microglial phagocytic activity, and demonstrate that REV‐ERBα deletion reduces amyloid plaque accumulation in 5XFAD mice. Our findings suggest that REV‐ERBs are important regulators of Aβ pathology and suggest that they may be a therapeutic target to delay AD progression.

## 2 RESULTS

### 2.1 Diurnal expression of circadian genes in vivo in microglia and macrophages

**Figure 1:**
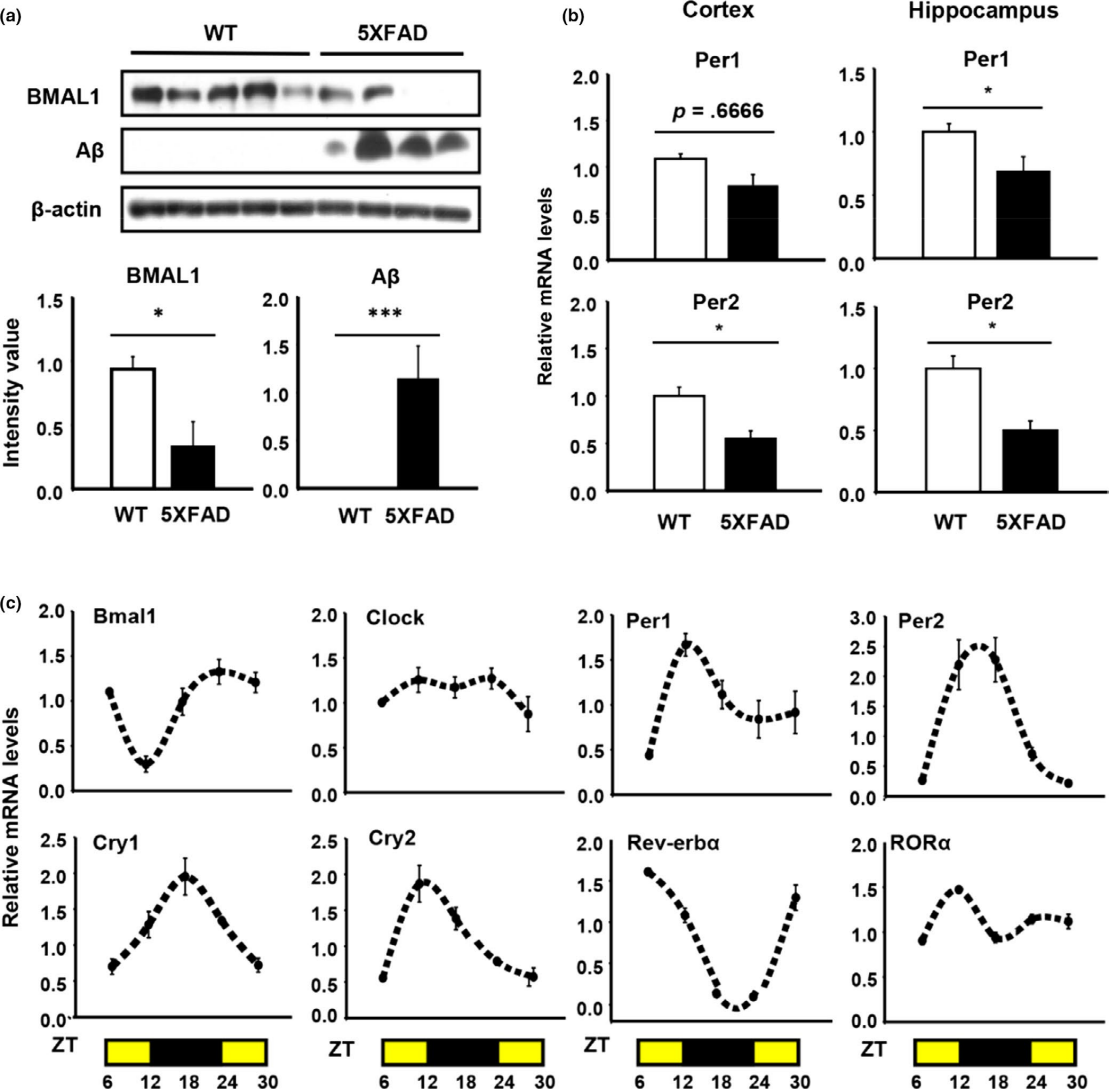
Patterns of circadian gene expression in murine peritoneal macrophages in vitro and microglia in vivo. (a) The expression of core clock protein, BMAL1, and Aβ in the cortex of WT and 5XFAD at 6.5 months. *p < .05, ***p < .001 compared to WT. (b) Comparing the mRNA levels of Per1/Per2 in the cortex and hippocampus of WT and 5XFAD. *p < .05 compared to WT. (c) The expression of several clock‐related genes in peritoneal macrophages is time‐dependent

To investigate whether circadian gene expression was disrupted in a mouse model of AD, we measured the level of BMAL1, a core clock gene, in 6.5‐month WT and 5XFAD mouse brain by Western blot. BMAL1 was severely attenuated in 5XFAD cortex compared with WT (Figure 1a). In addition, *Period1 (Per1)* and *Period2 (Per2)* were significantly dampened in 5XFAD cortex as well as in the hippocampus at the transcription levels (Figure 1b). Next, we initially confirmed that myeloid lineage cells possess molecular clock machinery in vivo prior to investigating the effect of circadian clock genes on microglial activity in AD. To test this, we isolated murine peritoneal macrophages at Circadian Time (CT) 6, 12, 18, 24, and 30. This revealed that, in peritoneal macrophages, the expression of several key clock components (Bmal1, Clock, Cry1, Cry2, Per1, Per2, Rev‐erbα, and RORα) dynamically oscillated in a time‐dependent manner (Figure 1c), in keeping with previous reports (Keller et al., 2009). In particular, the expression of *Bmal1*, which encodes a core clock protein, was lowest at CT12 and peaked at around CT24. To more directly investigate the diurnal expression of Bmal1 in microglia, we performed double immunohistochemical staining for the Bmal1 and microglial marker, Iba1 at CT12 and CT24 in mouse brain sections that included striatum. Similar to previous in vivo data (Figure 1c), Bmal1 expression was higher at CT24 than at CT12 in Iba1‐positive cells and dramatically decreased in 5XFAD mice, especially at ZT24 (Figure S1). Interestingly, the daily pattern of BMAL1 expression in microglia entirely reversed in the brain of 5XFAD mice compared to WT mice between ZT12 and ZT24 (Figure S1).

### 2.2 Regulation of Aβ uptake and clearance by clock proteins in BV‐2 microglia

**Figure 2:**
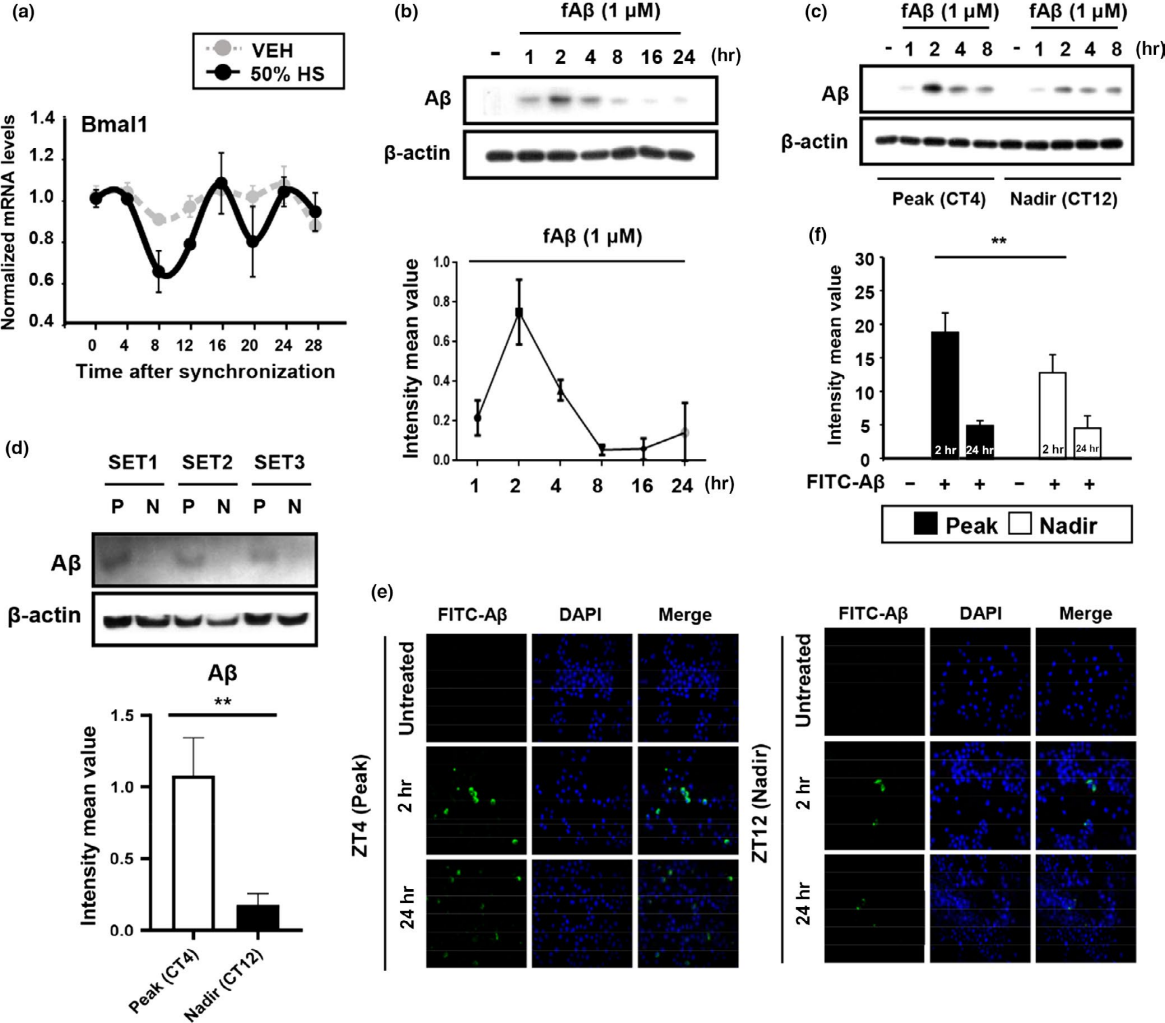
The phagocytic capacity of BV‐2 microglia varies with circadian gene expression. (a) The pattern of the clock gene *Bmal1* expression in BV‐2 cells. BV‐2 cells were synchronized with 50% horse serum (HS), and total RNA was extracted every 4 hr for 28 hr. (b) The rate of Aβ degradation in synchronized BV‐2 cells. The graph shows the densitometric quantification of the immunoblot bands. (c) fAβ_1‐42_ internalization was more efficient at circadian time (CT) 4 than at CT12. Representative Western blot and relative band densities of Aβ in BV‐2 cell lysates at different time points (1, 2, 4, and 8) after fAβ_1‐42_ treatment. (d) Total amount of engulfed Aβ in the cell lysate after 2 hr. We treated fAβ_1‐42_ (1 µM) in synchronized BV‐2 Cells at the different time point, Peak (CT4) and Nadir (CT12), respectively. **p < .01. (P: Peak, N: Nadir) (e) Representative fluorescent images of FITC‐fAβ_1‐42_‐positive cells over time (left) and (f) normalized fluorescence intensity values at CT4 and CT12 (right). BV‐2 cells were initially treated with 100 nM FITC‐fAβ_1‐42_. **p < .01

We then examined the expression of circadian genes in vitro using immortalized BV‐2 mouse microglial cells. BV‐2 cells were synchronized with 50% horse serum (HS) for 2 hr. Interestingly, synchronized BV‐2 cells expressed *Bmal1* in a biphasic manner that is not clearly circadian (Figure 2a). However, in order to test the effects of clock gene expression levels on Aβ uptake, we defined CT4 and CT12 as the peak and nadir times of *Bmal1* expression, respectively. To explore how the daily rhythms of gene expression affected microglial uptake of fAβ_1–42,_ we treated synchronized BV‐2 cells with fAβ_1–42_ (1 µM) at CT4 and CT12 and then analyzed the amount of fAβ_1–42_ in cell lysates. In synchronized BV‐2 cells, fAβ_1–42_ (1 µM) uptake was highest 2 hr after treatment (Figure 2b). Interestingly, we observed that microglia engulfed more fAβ_1–42_ at CT4 than at CT12 (Figure 2c,d). Using immunocytochemistry, we confirmed that more FITC‐Aβ_1–42_ (100 nM) was taken up by microglia at CT4 (Figure 2e). Thus, Aβ uptake by BV‐2 cells varies with time of day in parallel with Bmal1 expression.

**Figure 3:**
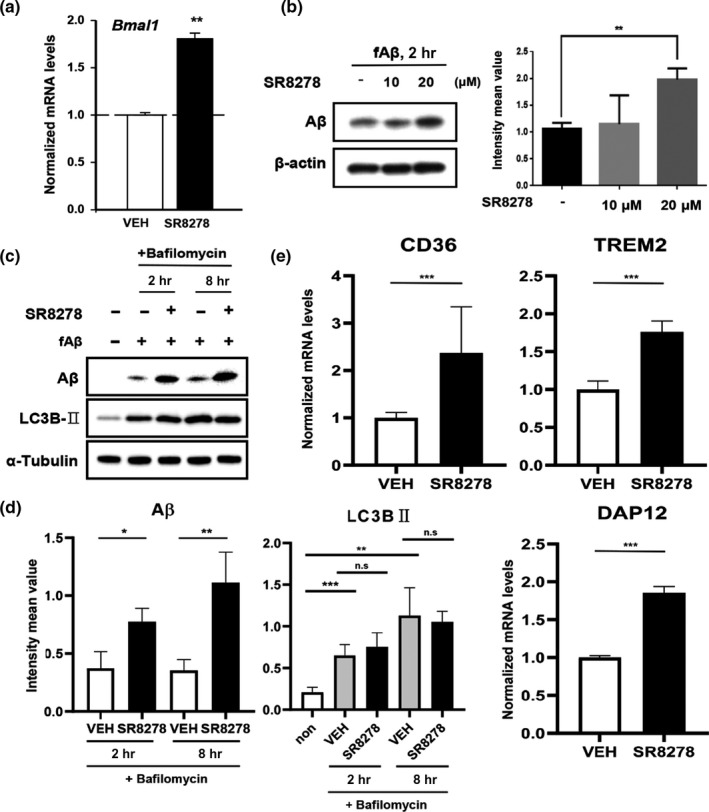
Inhibition of REV‐ERBs by SR8278 induces Bmal1 and other Aβ internalization‐related receptors and accelerates the Aβ uptake. (a) Effects of the REV‐ERBs antagonist, SR8278 (20 µM) on *Bmal1* expression. **p < .01. (b) SR8278 increased Aβ internalization. Synchronized BV‐2 cells were preincubated with SR8278 (10 µM, 20 µM) for 24 hr before treatment with fAβ_1‐42_ (1 µM) for 2 hr. **p < .01. (c‐d) Time‐dependent accumulation of Aβ in the cell lysate by SR8278 with LC3BII accumulation under the Bafilomycin‐treated conditions. Synchronized BV‐2 cells were preincubated with SR8278 (20 µM) or vehicle DMSO for 24 hr and added Bafilomycin (100 nM) for 1 hr. Aβ levels were measured at 2 and 8 hr after treatment. *p < .05, **p < .01 and ***p < .001 compared to vehicle‐treated group. Experiments were replicated three times. (e) Aβ internalization‐related receptors (CD36 and TREM2) and TREM2 adaptor protein (DAP12) were measured after SR8278 (20 µM) treatment. ***p < .001 compared to vehicle‐treated group

We then tested whether the pharmacological manipulation of the core circadian clock could alter fAβ_1‐42_ uptake. SR8278 is known to inhibit REV‐ERBα/β activity, thereby reducing repressive effects on Bmal1 and inducing Bmal1 expression (Kojetin, Wang, Kamenecka, & Burris, 2011). Moreover, Bmal1 drives expression of REV‐ERBα/β, suggesting that REV‐ERBs could control Aβ uptake in microglia downstream of Bmal1. As expected, SR8278 treatment (20 μM) upregulated *Bmal1* (Figure 3a) and increased fAβ_1–42_ uptake by BV‐2 cells relative to vehicle treatment in a dose‐dependent manner (Figure 3b). To verify that the effect of SR8278 was on Aβ uptake, not its degradation, BV2 cells were treated with a Bafilomycin 1A (Baf) which blocks autophagic flux. We measured engulfed fAβ_1–42_ levels in cell lysate after 2 and 8 hr under the Baf treatment. SR8278 again increased the amount of engulfed fAβ_1–42_ even when degradation was blocked (Figure 3c,d). This effect was more obvious after 8 hr fAβ_1–42_ treatment. In addition, SR8278 significantly increased Aβ internalization‐related receptors such as CD36 and TREM2, as well as the TREM2 adaptor gene DAP12 (Figure 3e). Altogether, these data indicate that in BV‐2 cells, alterations of circadian gene expression modulate fAβ_1–42_ uptake and that pharmacologic inhibition of REV‐ERBs increased fAβ_1–42_ uptake.

### 2.3 siRNA‐mediated REV‐ERB knockdown accelerates the fAβ_1–42_ uptake in primary microglia

**Figure 4:**
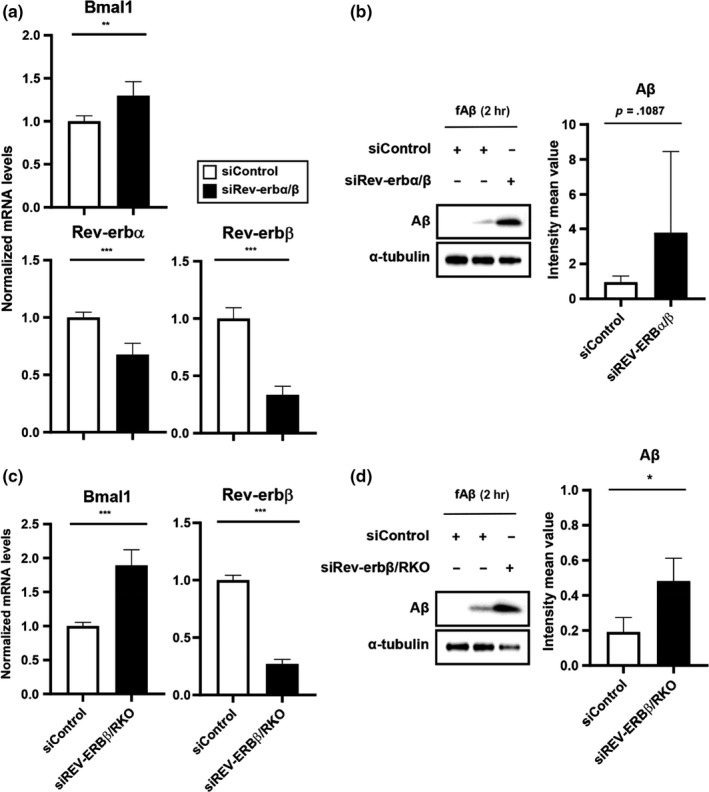
Knockdown of REV‐ERBα/β accelerates the microglial Aβ uptake in primary microglia. (a) mRNA Expression of Bmal1, Rev‐erbα, and Rev‐erbβ in siREV‐ERBα/β‐transfected WT mouse primary microglia. **p < .01, ***p < .001 compared to siControl‐transfected group. (b) Internalized Aβ levels in the cell lysate of siREV‐ERBα/β‐transfected WT mouse primary microglia and siControl‐transfected group, after 2 hr of Aβ exposure. (c) Expression of Bmal1 and Rev‐erbβ in siREV‐ERBβ‐transfected RKO mouse primary microglia. ***p < .001 compared to siControl‐transfected cells. (d) Internalized Aβ levels in the cell lysate of siREV‐ERBβ‐transfected RKO mouse primary microglia and siControl‐transfected cells. *p < .05 compared to siControl‐transfected cells

To confirm the enhancement of microglial fAβ_1–42_ uptake following REV‐ERBs inhibition, we measured amount of engulfed fAβ_1–42_ in primary mouse microglia using siRNA targeting both REV‐ERBs. We achieved a only partial knockdown of Rev‐erbα (35%) and Rev‐erbβ (60%) at the transcription levels, but it was adequate to induce increased expression of Bmal1 (Figure 4a). We found that fAβ_1–42_ uptake was induced in siREV‐ERBs transfected primary microglia but was not affected in cells transfected with control siRNA (Figure 4b). We also used siRNA to knockdown REV‐ERBβ levels in primary microglia from REV‐ERBα knockout (RKO) mice (Figure 4c) and then measured the levels of fAβ_1–42_ after 2 hr treatment. As we expected, fAβ_1–42_ uptake was increased in siREV‐ERBβ/RKO primary microglia compared with siControl‐transfected WT primary microglia (Figure 4d). From these results, we clearly suggest that microglial fAβ_1–42_ uptake was regulated REV‐ERBs‐dependent manner.

### 2.4 SR8278 upregulates P2Y_12_R expression and promotes M2 polarization

**Figure 5:**
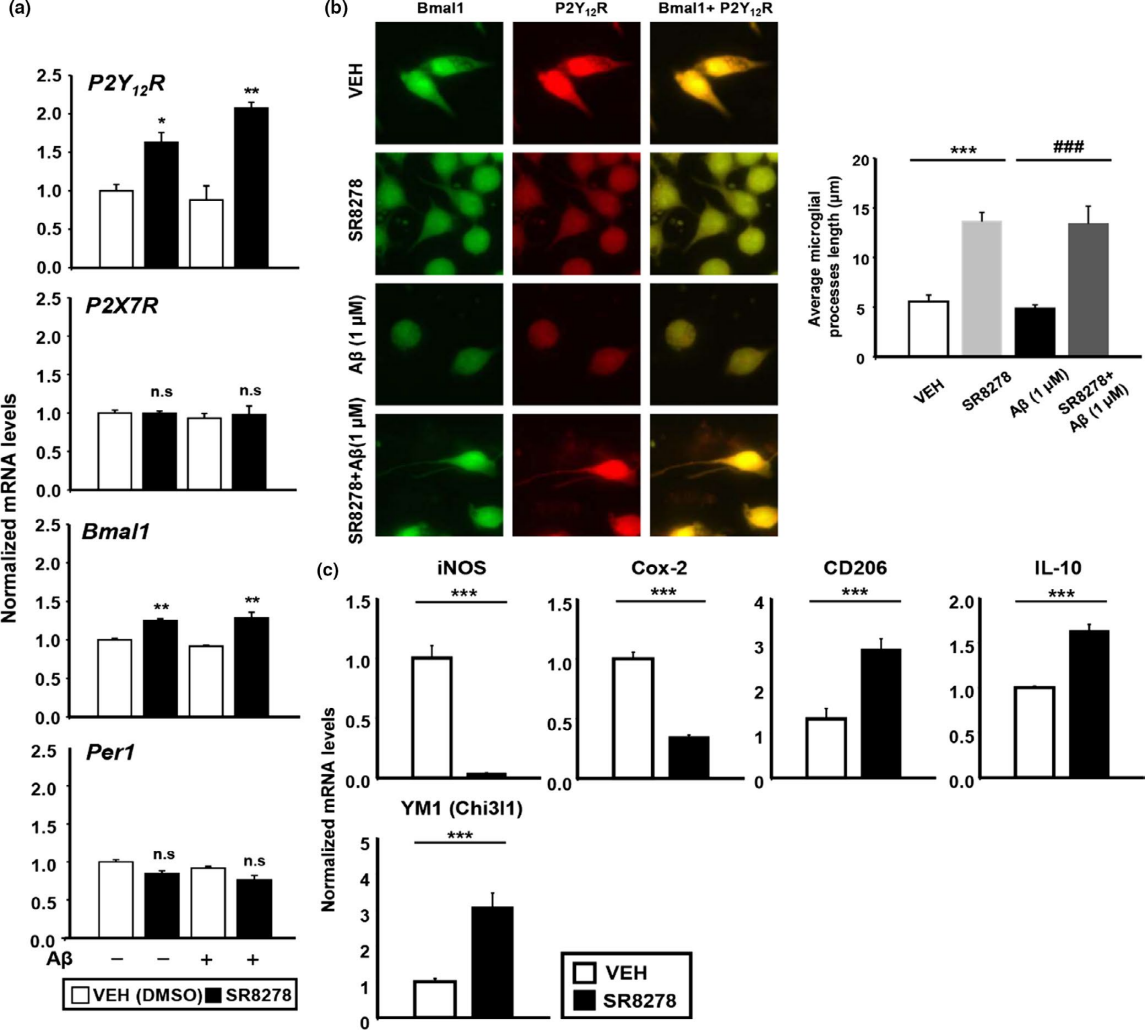
SR8278 induces microglial process extension and expression of P2Y_12_R and Bmal1. (a) In both the presence and absence of fAβ_1‐42_, SR8278 treatment significantly induced P2Y_12_R and Bmal1, but not P2X7R or Per1, in a dose‐dependent manner. Synchronized BV‐2 cells were pretreated with SR8278 (20 µM) for 24 hr before treatment with fAβ_1‐42_ (1 µM, 2 µM) for 2 hr. Each gene was analyzed using qPCR. *p < .05, **p < .01. (b) SR8278 (20 µM) recovered the fluorescence intensity of Bmal1 and P2Y_12_R and increased microglial process length in either the absence or presence of fAβ_1‐42_ (P2Y_12_R in red and Bmal1 in green). The graph shows the average length of the longest microglial processes from the 46 microglia in each group. ***p < .001 compared to the vehicle‐treated group and ^###^ *p* < .001 compared to the fAβ‐treated group. (c) The expression of M1‐type Markers (iNOS, Cox‐2) and M2‐type markers (CD206, IL‐10, YM‐1) after SR8278 (20 µM) treatment for 24 hr in BV‐2 cells was determined using qPCR. ***p < .001 compared to the vehicle‐treated group

Microglia express many purinergic receptors, including P2Y_12_R and P2X7R. These receptors, which regulate microglia process length, have been closely linked to circadian gene expression. Indeed, P2Y_12_R expression and P2X7R expression are directly modulated by Bmal1 and Per1, respectively (Hayashi, 2013; Nakazato et al., 2011). Therefore, we hypothesized that since SR8278 increases Bmal1 expression, it might regulate P2Y_12_R and P2X7R expression and subsequently alter microglial morphology. We first examined whether SR8278‐induced microglial activation was associated with changes in P2Y_12_R expression and P2X7R expression. To test this, we analyzed the expression of these receptors in BV‐2 cells using quantitative PCR (qPCR) and immunocytochemistry. Interestingly, SR8278 induced P2Y_12_R expression at the transcript level in both the presence and absence of fAβ_1–42_ (Figure 5a). It also induced the upregulation of *Bmal1* but not *Per1* (Figure 5a). We then examined how changes in P2Y_12_R expression affected microglial morphology by observing cells after SR8278 treatment in the presence or absence of fAβ_1–42_. This revealed that SR8278 significantly increased both microglial process length and P2Y_12_R expression (Figure 5b). Together, these data suggest that SR8278 increases the expression of P2Y_12_R in microglia, perhaps by regulating *Bmal1* expression. These effects may initiate microglial chemotaxis to promote fAβ_1–42_ internalization. We further investigated whether the elongation of microglial processes was induced when Bmal1 was at its peak (ZT24) in vivo using brain sectioning. As expected, microglial process length was higher at ZT24 than at ZT12 (Figure S2). However, it was dampened in 5XFAD mice (Figure 5c) along with Bmal1 downregulation (Figure S1).

Due to the high levels of microglia phagocytic activation induced by SR8278, we hypothesized that SR8278 would promote M2 microglial polarization, as M2 gene expression is associated with phagocytic activation. As expected, SR8278 dramatically increased the expression of M2 surface markers (*CD206*, *IL‐10*, and *YM‐1*) and decreased the expression of the M1 signature markers (*iNOS* and *Cox‐2*), indicating a shift toward M2 phenotype following SR8278 treatment (Figure 5d). These results suggest a role for REV‐ERBs on microglia morphology as well as their phenotype.

### 2.5 Loss of REV‐ERBα suppresses amyloid plaque pathology

**Figure 6:**
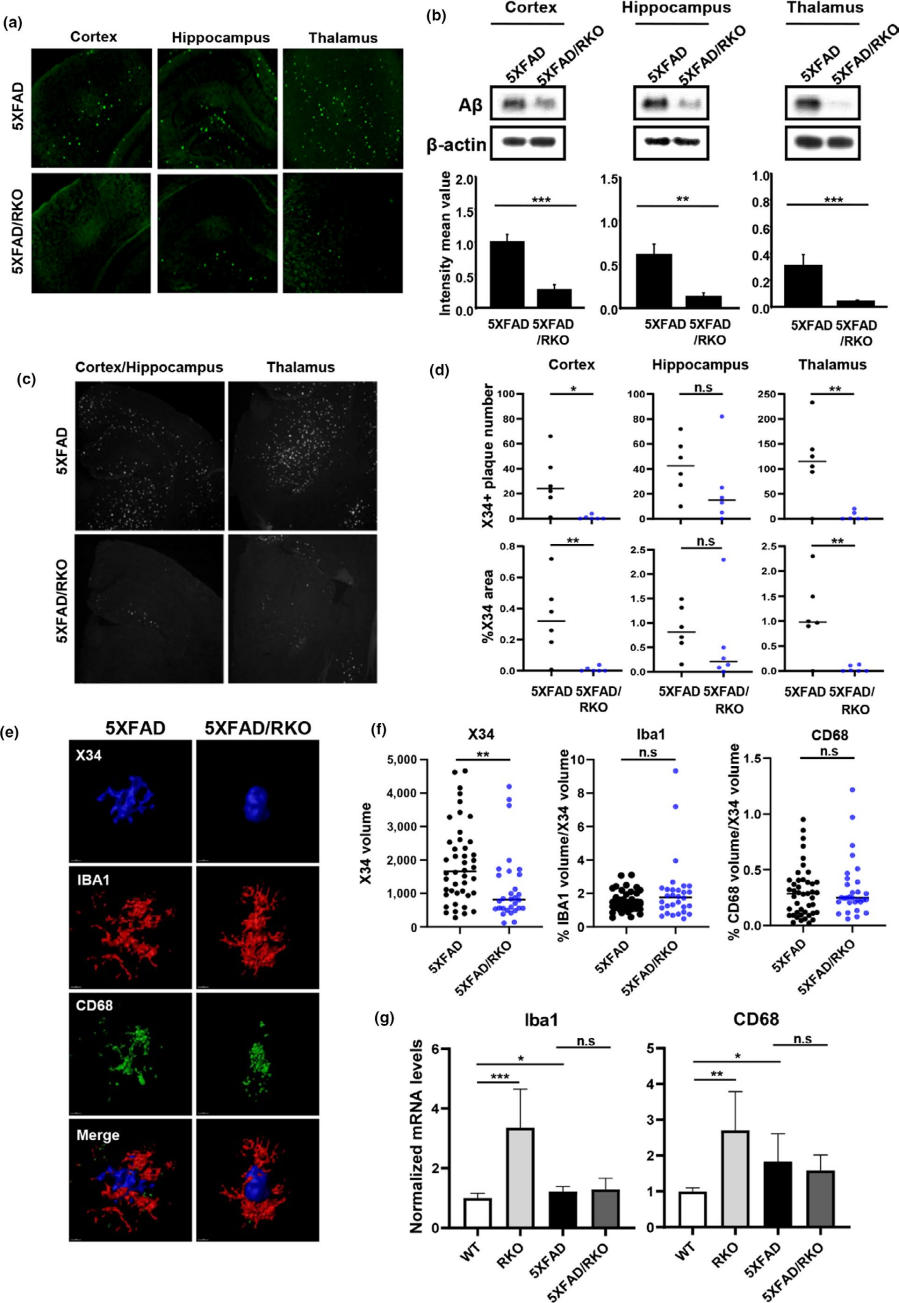
Deletion of REV‐ERBα mitigates amyloid plaque deposition in 5XFAD mice. (a) Representative image from thioflavin‐S staining of the brain sections including the cortex, hippocampus, and thalamus from 5XFAD and 5XFAD/REV‐ERα knockout (RKO) mice at 3.5 months. (*n* = 6–7 mice were analyzed per group). (b) Western blot analysis of Aβ peptide (4KDa) and β‐actin expression in each brain lysate. β‐actin was used as a loading control. **p < .01, ***p < .001 compared to the 5XFAD. (c) Representative image of X34 staining in the brain of 5XFAD and 5XFAD/RKO. (d) Quantification of X34‐positive plaque number and % area of X34 staining for each group mice brain using Image J. *p < .05, **p < .01 compared to the 5XFAD. (e) Representative images from confocal analysis of IBA1 and CD68 staining surrounding X34‐positive plaques in the cortex of 5XFAD and 5XFAD/RKO (X34 in Blue, IBA1 in Red, and CD68 in Green) (f) Quantification of X34‐positive plaque and plaque‐associated microglia (Iba1)/phagocytic microglia (CD68). Total volume of Iba1 and CD68 were normalized by X34 volume for each plaque. **p < .01 compared to the 5XFAD (*n* = 30–44 plaques) (g) mRNA expression of Iba1 and CD68 in the cortex of each group mice (WT, RKO, 5XFAD, 5XFAD/RKO). *p < .05, **p < .01, and ***p < .001

Because we observed a positive effect of REV‐ERBα/β knockdown or their antagonist SR8278 on the clearance of fAβ_1‐42_ in microglia in vitro, we suspected that REV‐ERBα‐depletion could mitigate amyloid plaque deposition in an AD mouse model. To test this, we crossed constitutive global REV‐ERBα KO mice with 5XFAD mice and analyzed plaque burden at 3.5 months old, an early plaque deposition time point. Using thioflavin‐S staining, we found that amyloid plaque in the brain including the cortex, hippocampus, and thalamus of 5XFAD mice was dramatically decreased by REV‐ERBα deficiency (Figure 6a). We also observed a striking reduction in total levels of Aβ using WB (Figure 6b) as well as a decrease in the number and size of plaques (Figure 6c–f) in the same brain regions of 5XFAD/RKO mice. Hippocampal X34 plaque burden did not reach statistical significance in 5XFAD/RKO mice due to a single mouse, but it was a strong trend toward a decrease in that region (Figure 6d). Since we observed a reduction in the number of plaques in REV‐ERBα‐deficient 5XFAD mice, we evaluated phagocytic microglia surrounding plaques in the brain. We stained for Iba1 to label microglia and CD68 to indicate microglial lysosomes, a marker of phagocytic activation. Plaque‐associated Iba1+/CD68+ microglia were not increased in 5XFAD/RKO compared with the cortex of 5XFAD (Figure 6e,f). This may be due to the markedly decreased number of plaques in the 5XFAD/RKO mice leading. In contrast, REV‐ERBα‐deficient mice without plaques showed high levels of Iba1 and CD68 at the transcription levels (Figure 6g). We suspect that phagocytic microglia activation caused by REV‐ERBα deletion causes Aβ clearance early in the disease stage and prevents plaques from ever forming, thereby also preventing plaque‐associated inflammation.

### 2.6 Loss of REV‐ERBα prevents plaques‐associated increases in DAM markers and synapse loss in 5XFAD mice

**Figure 7:**
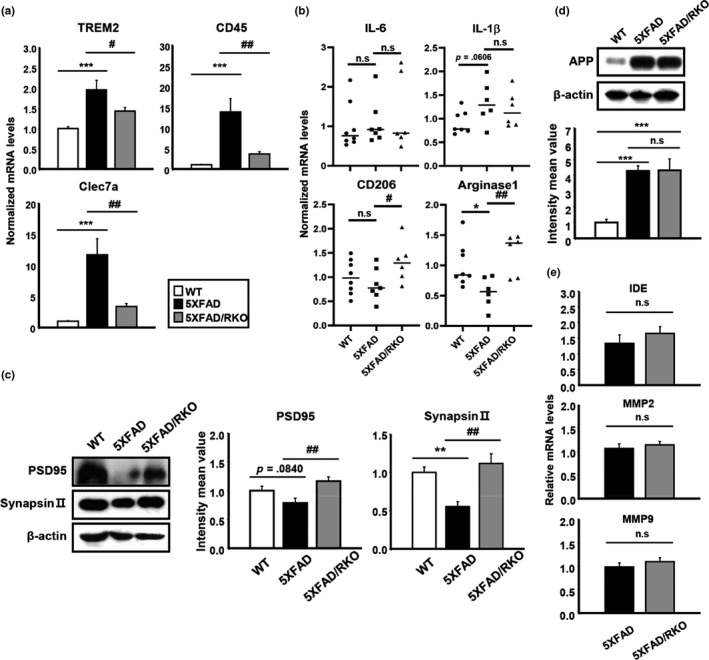
REV‐ERBα deletion in 5XFAD mice mitigates changes in DAM and synaptic markers and induces M2 microglial markers without alteration of APP processing. (a) mRNA expression of DAM markers including *TREM2*, *CD45*, and *Clec7a* and (b) pro‐inflammatory cytokines (*IL‐6* and *IL‐1β*) as well as the M2 surface markers (*CD206* and *Arginase1*) in the cortex of WT, 5XFAD, and 5XFAD/RKO. *p < .05, ***p < .001 compared to WT. ^#^ *p* < .05 and ^##^ *p* < .01 compared to the 5XFAD. (c) Western blot analysis of synaptic markers PSD95 and synapsin II in the cortex of all three different genotypes of mice. β‐actin was used as a loading control. **p < .01 compared to WT and ^##^ *p* < .01 compared to the 5XFAD. (d) Total amount of APP in the cortex of each group of mice by Western blot and (e) qPCR analysis of Aβ degradating enzymes (IDE, MMP2, and MMP9) from the same group of mice. ***p < .001 compared to WT

Because amyloid plaque deposition is associated with the accumulation of disease‐associated microglia (DAM) (Keren et al., 2017), we examined expression of the DAM markers *Trem2, Clec7a*, and *CD45* within the cortex of 5XFAD/RKO compared with the 5XFAD. All of these markers were significantly increased in 5XFAD but were increased to a lesser degree in RKO/5XFAD mice (Figure 7a). CD206 and Arginase 1 were both decreased in 5XFAD brain, while their levels were preserved in 5XFAD/RKO mice, suggesting that REV‐ERBα deletion can promote a phagocytic M2‐like state (Figure 7b), similar to our results in vitro (Figure 5d). Pro‐inflammatory cytokines IL‐6 and IL‐1β were unchanged in both 5XFAD and RKO/5XFAD at this young age (Figure 7b). We further observed a decrease in synaptic proteins (Synapsin and PSD95) in 5XFAD cortex which was rescued in 5XFAD/RKO mice (Figure 7c). It is likely that the diminished plaque burden in 5XFAD/RKO mice is what drives these changes in DAM marker expression and synaptic protein levels, though direct effects of REV‐ERBα on plaque‐related microglial changes cannot be excluded.

To ensure that these results were not due to differences in transgene expression, we examined the levels of genes and proteins involved in Aβ synthesis and degradation. Western blot analysis showed no differences between 5XFAD and 5XFAD/RKO in APP protein (Figure 7d), and transcript levels of Aβ‐degradating enzymes including IDE, MMP2, and MMP9 showed no significant changes between the two genotype (Figure 7e), suggesting that REV‐ERBα depletion did not alter APP expression and processing. Together, our findings indicate that REV‐ERBs have important role for Aβ clearance, likely via microglia, leading to diminished plaque accumulation in REV‐ERBα‐deficient mice.

## 3 DISCUSSION

Our study is the first to show that the microglial phagocytosis of Aβ undergoes circadian regulation. Herein, we demonstrated that the pharmacological inhibition of circadian repressor REV‐ERBα/β using SR8278 enhanced microglial Aβ phagocytosis activity and increased Bmal1 expression as well as induction of the Aβ internalization‐related receptors, CD36 and TREM2. Furthermore, we observed induction of P2Y_12_R, a microglia‐specific purinergic receptor, by SR8278 treatment, and found that SR8278 also led to M2 polarization in vitro and in vivo. Genetic knockdown of REV‐ERBs also enhanced Aβ uptake by microglia, and global deletion of REV‐ERBα strikingly reduced amyloid plaque burden without alteration of APP processing enzymes and amyloid precursor protein (APP) in the brain of 5XFAD mice. Disease‐associated microglia markers (DAMs) including Clec7a, CD45, and TREM2 were increased in 5XFAD mice but virtually returned to normal levels when REV‐ERBα was deleted. Ultimately, our results strongly suggest that REV‐ERBs inhibition could be considered as a therapeutic strategy for enhancing microglia‐mediated Aβ degradation and limiting amyloid plaque deposition in AD.

Numerous studies have suggested that the circadian system plays a pivotal role in neurodegenerative/neuroinflammatory diseases such as AD and Parkinson's disease (Musiek & Holtzman, 2016). Indeed, sleep and circadian dysfunction may manifest very early in AD progression (Musiek et al., 2018). Furthermore, chronic sleep deprivation increases amyloid plaque deposition (Kang et al., 2009), while sleep augmentation induced by the genetic deletion of orexin strongly suppresses amyloid plaque formation in AD mice (Roh et al., 2014). Moreover, disruption of the circadian system by deletion of Bmal1 accelerates plaque accumulation in APP/PS1 mice (Kress et al., 2018), though the mechanisms remain unclear. Despite increasing evidence that molecular clockwork exists in neuroglia, including microglia (Fonken et al., 2015; Jackson, 2011), the role of the microglial circadian system in amyloid clearance remained largely unknown. Our study shows that the core clock protein Bmal1 was more highly expressed at ZT24 than at ZT12 in murine microglia and this was completely reversed in 5XFAD mice (Figure S1), suggesting microglial clock disruption in this amyloidosis model. We also demonstrate a time‐of‐day dependence of microglial Aβ uptake, indicating that the microglial molecular clock machinery can be a key regulator of microglial activity in AD. Further studies are needed to explore the effect of AD pathology on microglial circadian clocks and the mechanisms by which the clock regulates microglial phagocytic function.

Perhaps our most important finding was that suppression of REV‐ERBα/β enhanced microglial Aβ phagocytosis in vitro and mitigate plaque deposition in 5XFAD mice in vivo. We demonstrated using REV‐ERBs antagonist, SR8278 and siRNA‐mediated knockdown experiments in vitro, as well as genetic manipulation of REV‐ERBα in vivo. SR8278 induced Bmal1 expression and accelerated microglial Aβ uptake even when lysosomal degradation was blocked with Bafilomycin A1 (Figure 3c), leading to an increase in the Aβ endocytosis‐related receptors CD36 and TREM2 (Figure 3d). Microglia cells express diverse receptors that cooperate in the recognition, internalization, phagocytosis, and clearance of Amyloid‐β, as well as the inflammatory response (Doens & Fernández, 2014). Among them, CD36/TLR4/TREM2 were considered as recycling receptors for Aβ phagocytosis and necessary factors for the LC3‐associated endocytosis (LANDO) pathway (Heckmann et al., 2019.). We suspect that REV‐ERBs activity might participate in LANDO via modulating the expression of receptors in microglia. TREM2 is a well‐characterized Aβ receptor that participates in Aβ endocytosis and elimination and can help glia‐mediated synaptic engulfment in neurodevelopment (Jay et al., 2019; Zhong et al., 2019). Interestingly, SR8278 significantly increased the expression of DAP12 which is considered as TREM2 adaptor in microglia, as well as induced TREM2 levels, indicating that SR8278 could propagate TREM2 downstream signaling in microglia. TREM2 induction was also seen following REV‐ERBα deletion in another paper (Griffin et al., 2019). Moreover, numerous studies support that TREM2 has critical role on tauopathy and amyloid pathology (Leyns et al., 2019). Thus, we suspect that REV‐ERBs could be a potent candidate for AD therapy targeting tau. However, it still remains to be seen how REV‐ERBs impact tau spreading/propagation. Taken together, our results suggest that pharmacological inhibition of REV‐ERBs may improve Aβ pathology through activating the microglial phagocytic activity in patients with AD.

Our data suggest that SR8278 may enhance microglial phagocytosis of Aβ by modulating P2Y_12_R expression. Microglia are sensitive to environmental changes and can immediately transform their morphology in response to purinergic receptor activation (Koizumi et al., 2013). Specifically, microglia that are initially highly branched or ramified can undergo process extension and increase P2Y_12_R expression. Recent studies have shown that cortical microglia rhythmically express P2Y_12_R throughout the day (Hayashi, 2013). This suggests that molecular clockwork may regulate microglial phagocytic behavior by modulating purinergic receptor expression, which could further accelerate the clearance of Aβ aggregates. In this study, we showed that SR8278 enhanced P2Y_12_R expression, thereby increasing microglial process length and enabling the phagocytosis of Aβ aggregates (Figure 5). Another purinergic receptor subtype—P2X7—is selectively upregulated by ATP‐induced *Per1* expression (Nakazato et al., 2011), but was unaffected by SR8278 (Figure 5a). In addition, the process length of microglia in 5XFAD mice (Figure 5c) exhibited a marked shortening at ZT24 with the reduction in Bmal1 (Figure S1), indicating that lower Bmal1 expression or reduced functioning may be specifically associated with microglia morphology and activity. Altogether, these results suggest that SR8278 modulates P2Y_12_R expression in microglia, perhaps by inducing Bmal1, and this may influence microglial morphology and Aβ uptake.

Given the effects of SR8278 on purinergic receptor expression and process length, it is possible that SR8278 promotes M2‐like microglial polarization. Recent several studies and researchers suggest that promoting the differentiation toward the neuroprotective M2 polarization is protective in models of neurodegenerative diseases and traumatic brain injury (Song & Suk, 2017). In our studies, SR8278 dramatically increased M2 type markers such as *CD206*, *IL‐10*, and *YM1 *in vitro as well as in vivo (Figurea 5d and 7b), indicating that it may further promote a phagocytic microglial phenotype. Moreover, the previous report suggests that autophagy activation can accelerate M2 microglia polarization under both basal and inflammatory conditions (Jin et al., 2018), and REV‐ERBs has been linked to regulation of autophagy (Woldt et al., 2013). From these results, we suspect that SR8278 might induce autophagy by suppressing REV‐ERB function, promoting Aβ clearance and M2‐like polarization.

Consistently, our study also showed that lower DAM markers which reflect “bad microglia” accompanied by more M2 microglia in REV‐ERBα‐deficient 5XFAD mouse brain. These data imply that modulating the REV‐ERBs activity can improve the brain damage via releasing the protective factors from M2 microglia. We observed that REV‐ERB inhibition/deletion alters microglial activation state to promote the removal of Aβ both in vitro and in vivo. Interestingly, REV‐ERBα‐deficient mice highly expressed Iba1 and CD68 as markers of microglia and phagocytic microglia, respectively, in the brain (Figure 6g). A previous report also showed significantly increased Iba1 and CD68 expression in hippocampal microglia of REV‐ERBα knockout mice (Griffin et al., 2019). Thus, we suspect that high levels of phagocytic microglia, evoked by REV‐ERBα depletion, are likely responsible for the marked decrease in plaque accumulation in RKO/5XFAD mice. Because these mice accumulate less plaque, there is a concomitant decrease in DAM microglial markers, as well as plaque‐related synapse loss (Figure 7c). However, it is possible that REV‐ERBs inhibition might directly promote synapse survival and limit DAM microglial marker expression independently of its effect on plaque burden, perhaps by promoting M2‐like polarization. Furthermore, because global, constitutive REV‐ERBα mice were used in our study with 5XFAD mice, we cannot exclude important contributions of cell types other than microglia, as REV‐ERBs likely play important roles in neurons and other brain cell types. However, we did not observe changes in APP processing or other Aβ metabolic enzyme expression. Future studies in cell type‐specific REV‐ERBα KO mice will be needed to address these possibilities in more detail.

Our studies investigating the REV‐ERBs antagonist SR8278 to definitively demonstrate the role of REV‐ERBs in microglial activation for Aβ clearance. Our data reveal that the inhibition of REV‐ERBs effectively enhanced microglial phagocytosis in vivo and in vitro and also selectively increased P2Y_12_R expression in microglia, suggesting that SR8278 can modulate microglial process motility and promote M2‐like polarization. In vivo, REV‐ERBα strongly suppressed plaque accumulation and downstream Aβ toxicity in 5XFAD mice. Ultimately, our results strongly suggest that the circadian system intimately controls microglial activation, potentially though REV‐ERBs regulation, and it has therapeutic implications for a number of neurological disorders.

## 4 EXPERIMENTAL PROCEDURES

### 4.1 Animals

5XFAD and REV‐ERBα knockout (KO) mice were purchased from Jackson Laboratories. REV‐ERBα KO mice have a β‐geo cassette replacing part of exon 2, all of exons 3–5, and part of exon 6 of the nuclear receptor subfamily 1, group D, member 1 (Nr1d1) gene, and abolishing gene function. To generate REV‐ERBα deficient 5XFAD mice, 5XFAD mice were bred with REV‐ERBα KO mice. Each group of mouse was housed in a different cage and was maintained at a constant ambient temperature (22 ± 1°C) with a 12:12 hr light‐dark cycle and free access to water and food. All procedures were approved by the Institutional Animal Care and Use Committee of the Asan Institute for Life Sciences in Seoul, Korea.

### 4.2 Reagents

SR8278 (#554718; Thermo Fisher Scientific) was dissolved in dimethylsulfoxide (DMSO). Solutions were aliquoted to avoid freeze‐thawing and stored at −80°C. Lipopolysaccharide (#L3024) was purchased from Sigma‐Aldrich.

### 4.3 Synthesis of fibrillar Aβ_1‐42_

Aβ_1‐42_ (#H1368; Bachem) and fluorescein isothiocyanate (FITC)‐conjugated Aβ_1‐42_ (#M2585; Bachem) were dissolved in DMSO to a final concentration of 500 µM (based on the original Aβ_1–42_ monomer concentration) and stored at −80°C. Before use, fibrillar Aβ_1–42_ (fAβ_1‐42_) was preincubated at 37°C for 24 hr in Dulbecco's modified Eagle's medium (DMEM) with high glucose (#21013024; Life Technologies). These compounds were then diluted 1:10 to a final concentration of 50 µM. FITC‐Aβ_1‐42_ was always kept in the dark.

### 4.4 Plaque staining

#### 4.4.1 Thioflavin‐S staining

Staining was performed using thawed fresh‐frozen sections postfixed in 4% paraformaldehyde (PFA). Free‐floating brain sections were washed with 1× phosphate‐buffered saline (PBS). The sections were soaked in 1% thioflavin S solution for 8 min. Subsequently, samples were washed with 70% ethyl alcohol (EtOH) for 5 min and were then washed twice with PBS. Sections were mounted using a fluorescence mounting medium (#S3023; Dako) after minimal drying.

#### 4.4.2 X34 staining

Free‐floating brain sections were washed three times with 1XPBS and incubated with 0.25% Triton X‐100 in 1XPBS for 30 min at RT. The sections were stained with X34 staining buffer (1:3,000) for 20 min and then washed three times with X34 wash buffer (40% EtOH in 1XPBS) for 2 min at RT. Sections were mounted using fluorescence mounting medium (#S3023; Dako) after two times of wash with 1XPBS for 5 min.

### 4.5 Cell culture

Murine‐immortalized microglial BV‐2 cells were grown in DMEM supplemented with 5% fetal bovine serum (#10082147; Life Technologies) and 100 U/ml penicillin and streptomycin (#15140122; Life Technologies). All cells were maintained at 37°C in a humidified atmosphere with 5% CO_2_.

### 4.6 Isolation of peritoneal macrophages

Wild‐type mice were injected intraperitoneally with 3 ml of 1× PBS at ZT 6, 12, 18, 24, and 30 (*n* = 3 per point in time). Three hours later, primary macrophages were collected from the peritoneal cavities of the anesthetized animals using 10‐ml syringes. Macrophages were obtained by centrifugation at 400 *g* and 4°C. Following washing with PBS twice, each pellet was analyzed using qPCR.

### 4.7 Isolation of primary microglia

Microglia were isolated from mixed glial that were obtained from the cerebral cortex of postnatal days 1–3 (P1‐3) mice. Cortices were dissected stripped of meninges with cold DMEM and trypsinization with 0.05% trypsin‐EDTA at 37°C for 10 min. Cells were suspended with complete media containing GM‐CSF (5 ng/ml) after centrifugation for 5 min and replated coated with PDL. Floating microglia were collected from the mixed glial cultures by shaking the flask at 225 rpm for 2 hr after 10 days.

### 4.8 siRNA transfection

Primary microglia were transfected with siRNA using lipofectamine RNAiMAX (Life Technologies) in OptiMEM (Life Technologies) according to the manufacturer's instructions. siRNAs targeting mouse Nr1d1, Nr1d2, and scramble were obtained from Dharmacon (Lafayette, CO). A siRNA to RNAiMAX ratio of 1:1.25 was used, and 40 pmol of siRNA (2.5 μL of 20 μM stock) was added to each well of a 12 well plate. Media was changed after 7 days.

### 4.9 RNA preparation and qPCR analysis

Total RNA was extracted from cells using a NucleoSpin RNA kit (#740955.250; Macherey‐Nagel) according to the manufacturer's instructions. RNA concentrations were determined using a Nanodrop ND 1000 spectrophotometer. cDNA was then synthesized using approximately 1 µg of RNA and the ReverTra Ace qPCR RT Kit (#FSQ‐101; Toyobo) according to the manufacturer's instructions. qPCR was performed on diluted cDNA samples using either iQ SYBR Green Supermix (#1708882; Bio‐rad) or TaqMan primers and maters mix (Thermo) with a StepOnePlus RT‐PCR system. Melting curve analysis confirmed the specificity of each SYBR Green reaction. The PCR primer sequences are listed in Table 1.

**Table 1:** Primer sequences used for quantitative PCR

Gene	Primer sequences
Bmal1	F: 5'‐CCT AAT TCT CAG GGC AGC AGA T‐3' R: 5'‐TCC AGT CTT GGC ATC AAT GAG T‐3'
Clock	F: 5'‐TTG CTC CAC GGG AAT CCT T‐3' R: 5'‐GGA GGG AAA GTG CTC TGT TGT AG‐3'
Cry1	F: 5'‐AAA AAT TCA CGC CAC AGG AG‐3' R: 5'‐CGA ATG AAT GCA AAC TCC CT‐3'
Cry2	F: 5'‐GCT CCC AGC TTG GCT TGA‐3' R: 5'‐TGT CCC TTC CTG TGT GGA AGA‐3'
Per1	F: 5'‐GTG TCG TGA TTA AAT TAG TCA G‐3' R: 5'‐ACC ACT CAT GTC TGG GCC‐3'
Per2	F: 5'‐GCG GAT GCT CGT GGA ATC TT‐3' R: 5'‐GCT CCT TCA GGG TCC TTA TC‐3'
Rev‐erbα	F: 5'‐AGC TCA ACT CCC TGG CAC TTA C‐3' R: 5'‐CTT CTC GGA ATG CAT GTT GTT C‐3'
RORα	F: 5'‐GCA CCT GAC CGA AGA CGA AA‐3' R: 5'‐GAG CGA TCC GCT GAC ATC A‐3'
P2Y12R	F: 5'‐ CAC AGA GGG CTT TGG GAA CTT A ‐3' R: 5'‐ TGG TCC TGC TTC TGC TGA ATC ‐3'
P2X7R	F: 5'‐ TGT GTG CAT TGA CTT GCT CA ‐3' R: 5'‐ CTT GCA TTT TCC CAA GC ‐3'
COX‐2	F: 5'‐GCA AAT CCT TGC TGT TCC AAC C‐3' R: 5'‐GGA GAA GGC TTC CCA GCT TTT G‐3'
CD206	F: 5'‐AGT TGG GTT CTC CTG TAG CCC AA‐3' R: 5'‐ACT ACT ACC TGA GCC CAC ACC TGC T‐3'
Nrf‐2	F: 5'‐CAA GAC TTG GGC CAC TTA AAA GAC‐3' R: 5'‐AGT AAG GCT TTC CAT CCT CAT CAC‐3'
CD36	F: 5'‐ TCG GAA CTG TGG GCT CAT ‐3' R: 5'‐ CCT CGG GGT CCT GAG TTA TAT TTT C ‐3'
TREM2	F: 5'‐ TGG GAC CTC TCC ACC AGT T ‐3' R: 5'‐ GTG GTG TTG AGG GCT TGG ‐3'
DAP12	F: 5'‐ GAT TGC CCT GGC TGT GTA CT ‐3' R: 5'‐ CTG GTC TCT GAC CCT GAA GC ‐3'
CD45	F: 5'‐ TCA GCA CTA TTG GTA GGC TCC ‐3' R: 5'‐ ATG GTC CTC TGA ATA AAG CCC A ‐3'
Clec7a	F: 5'‐ GTG CAG TAA GCT TTC CTG GG ‐3' R: 5'‐ TCC CGC AAT CAG AGT GAA G ‐3'
Arginase1	F: 5'‐ TCA CCT GAG CTT TGA TGT CG ‐3' R: 5'‐ TTC CCA AGA GTT GGG TTC AC ‐3'
YM1	F: 5'‐ ACC CCT GCC TGT GTA CTC ACC T ‐3' R: 5'‐ CAC TGA ACG GGG CAG GTC CAA A ‐3'
IL‐10	F: 5'‐ AAT TCC CTG GGT GAG AAG CTG ‐3' R: 5'‐ TCA TGG CCT TGT AGA CAC CTT G ‐3'
IDE	F: 5'‐ GAA CGA TGC CTG GAG ACT CTT ‐3' R: 5'‐ TTC CCT TAC GTC GAT GCC TTC ‐3'
MMP2	F: 5'‐ CAA GTT CCC CGG CGA TGT C ‐3' R: 5'‐ TTC TGG TCA AGG TCA CCT GTC ‐3'
MM9	F: 5'‐ GAG ACG GGT ATC CCT TCG AC ‐3' R: 5'‐ TGA CAT GGG GCA CCA TTT GAG ‐3'
GAPDH	F: 5'‐CAT GGC CTT CCG TGT TCC TA‐3' R: 5'‐CCT GCT TCA CCA CCT TCT TGA‐3'

### 4.10 Immunoblot

Samples were harvested with a PRO‐PREP protein extraction kit (#17081; iNtRON) supplemented with phosphatase inhibitor cocktail 2 (#P5726; Sigma‐Aldrich) and centrifuged to remove cell debris. The concentrations of the prepared protein samples were determined using Bradford assays. Protein samples were separated by electrophoresis on 10%–15% sodium dodecyl sulfate–polyacrylamide gels and then transferred electrophoretically to polyvinylidene difluoride membranes. The membranes were blocked with 5% skim milk and then washed with PBS containing 0.05% Tween® 20. The membranes were then gently agitated and incubated at 4°C overnight with the following primary antibodies: anti‐Aβ (1:500, 6E10; #SIG‐39340 or 1:1,000, 82E1; IBL‐America), anti‐β‐actin (1:1,000, AC‐15, #A5441; Sigma), and anti‐α‐tubulin (1:1,000, T5168; Merck). The following day, the membranes were washed and then incubated with horseradish peroxidase‐labeled anti‐rabbit or anti‐mouse secondary antibodies for 40 min at room temperature. Subsequently, membrane‐bound horseradish peroxidase‐labeled antibodies were detected using an enhanced chemiluminescence detection system including the Pierce ECL Western Blotting Substrate (#32106; Thermo Fisher Scientific). Densitometric quantification of the bands was conducted using ImageJ (Image Processing and Analysis in Java; National Institutes of Health). Protein levels were normalized to β‐actin or α‐tubulin for quantification.

### 4.11 Immunocytochemistry

Cells were seeded onto 24‐well plates with poly‐l‐lysine‐coated coverslips and fixed with 4% paraformaldehyde (#A2025; Biosesang) for 15 min. Subsequently, each well was washed with PBS and then incubated in blocking medium (3% bovine serum albumin [Probumin, #821006; Millipore] in PBS) for 30 min at room temperature. The samples were incubated for 1 hr with the following primary antibodies diluted in PBS with 0.1% Triton X‐100 and 10% HS: rabbit P2Y_12_R (1:500, NBP1‐78249, #64805; Novus Biologicals), mouse Bmal1 (1:200, B‐1, #sc‐365645; Santa Cruz Biotechnology), and rabbit Iba1 (1:1,000, #016‐20001; Wako Chemicals). After washing them three times with PBS, the cells were incubated with secondary Alexa Fluor™ 488‐ and 594‐conjugated goat anti‐mouse and goat anti‐rabbit antibodies (Jackson ImmunoResearch Laboratories) diluted to 1:500 in PBS with 0.1% Triton X‐100 and 10% HS for 30 min at room temperature. The nuclei were stained with 4′,6‐diamidino‐2‐phenylindole (DAPI) for 10 min, and then, the cells were washed with PBS and mounted using a fluorescence mounting medium (#S3023; Dako).

### 4.12 Immunohistochemistry

Fixed hemispheres of the mouse brains were cut into 35‐μm sections (coronal sections) using a Leica VT1000S vibratome. Free‐floating sections were washed with PBS three times for 5 min, blocked with 3% bovine serum albumin for 30 min, and finally incubated with primary antibodies diluted in PBS [(rabbit Iba1, 1:1,000, #016‐20001; Wako Chemicals), (rat CD68, 1:150, MCA1957; Bio‐rad)] overnight at 4°C. Incubated slices were then washed with PBS three times for 5 min, incubated for 2 hr at room temperature with a secondary antibody (1:400; Jackson Laboratories) in PBS, and then washed with PBS three times for 5 min at room temperature. Cells were stained with DAPI and mounted using fluorescence mounting medium (#S3023; Dako). Fluorescent images were taken with a Zeiss Axio Observer Z1 microscope and processed using AxioVision 4.8.2.

### 4.13 Confocal Imaging and 3D Reconstructions

Images were acquired using a LSM 710 Confocal microscope (Zeiss) and the ZEN 2011 software package. Laser and detector settings were maintained constant for the acquisition of each immunostaining. Z stacks were obtained from 30‐μm‐thick sections using Colocalization analysis, and 3D reconstructions were created using Imaris 8 software. For quantification of plaque volume, images were imported to Fiji software (Image J) and data channels were separated (image/color/split channels). The volume of IBA1‐ and CD68‐positive microglia around plaques were measured in the Cortex over the length of layers 3–5 using Image J.

### 4.14 Statistical analysis

For the statistical analysis, Student's *t* tests (comparing two groups) or one‐way analyses of variance (ANOVAs) with Tukey post hoc tests were performed using GraphPad Prism software 8 and Sigma Plot 8.0. Differences were considered significant at *p < .05, **p < .01, and ***p < .001. All experiments were replicated six times and are shown as the mean ± *SEM*.

## CONFLICT OF INTEREST

None declared.

## AUTHOR CONTRIBUTIONS

J.L designed the study, performed the experiments, analyzed the data, and wrote the manuscript. DE.K performed the experiments. P.G and P.W.S contributed materials/analytic tools. SY.Y, E.S.M, and DH.K supervised the study design and revised the manuscript. All authors read and approved the final manuscript.

## Data Availability

All data supporting the findings of this study are available within the paper and its extended data files.
