# Supplementary material for: Inhibition of REV‐ERBs stimulates microglial amyloid‐beta clearance and reduces amyloid plaque deposition in the 5XFAD mouse model of Alzheimer’s disease
Source: Aging Cell. 2019 Dec 4;19(2):e13078. doi: 10.1111/acel.13078 (PMC6996949; doi:10.1111/acel.13078)
Supplement: Supplementary file 1 [file ACEL-19-e13078-s001.pdf]

Figure S1. Lee J et. al.

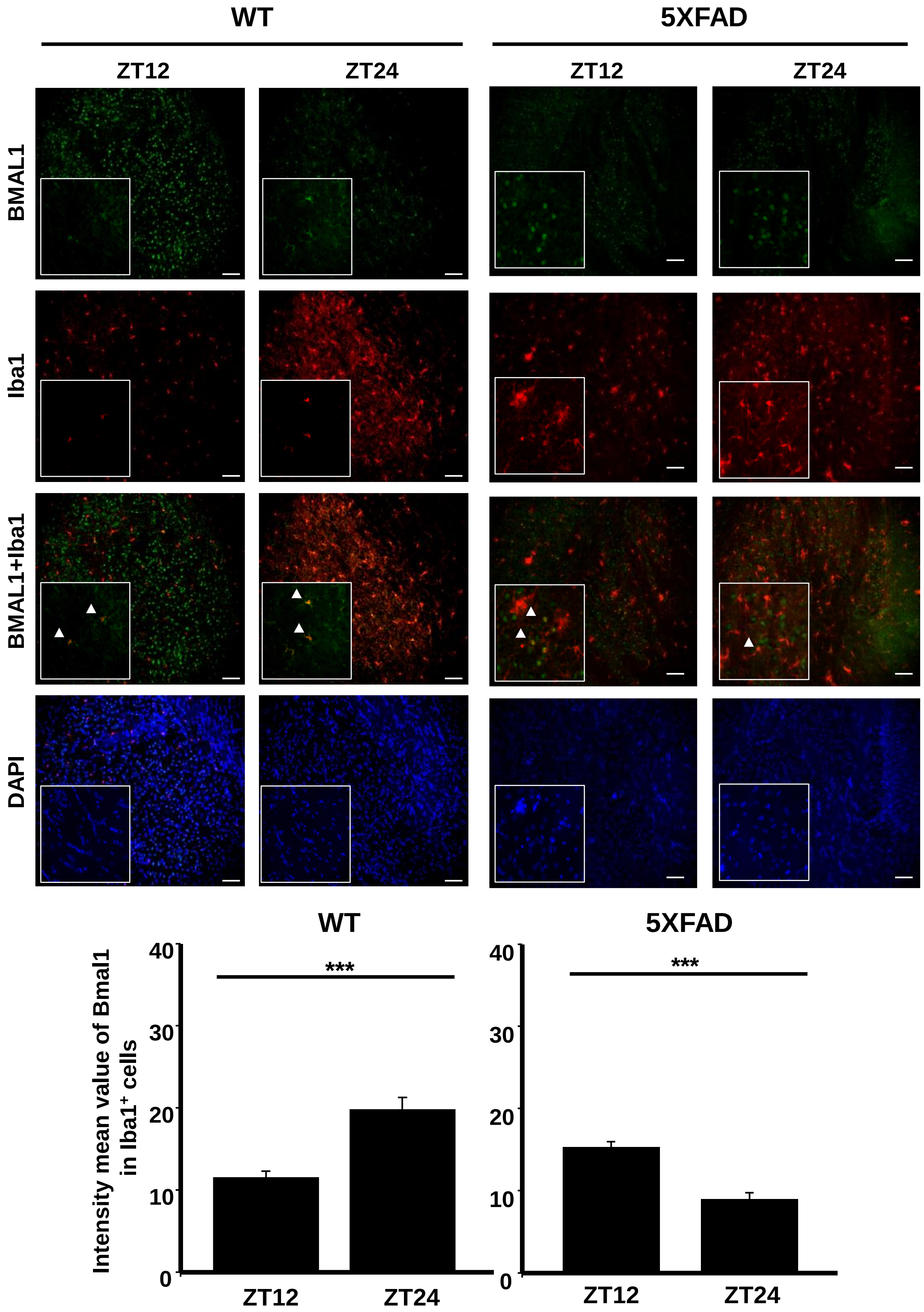

Figure S1. Diurnal expression of Bmal1 in microglia entirely reversed in the brain of 5XFAD mice compared to WT mice between ZT12 and ZT24. Representative images of immunostaining for Bmal1 and Iba1 in the Striatum of a wild-type mouse and 5XFAD mouse (DAPI in blue, Iba-1 in red, and Bmal1 in green) at Zeitgeber time (ZT) 12 and 24. Arrowheads indicate cells co-labeled with Bmal1 and Iba-1. The insets show higher magnification images. The mean total intensity of Bmal1 labeling in $Iba1^{+}$ cells at ZT12 and ZT24. ***$P < 0.001$.

Figure S2. Lee J et. al.

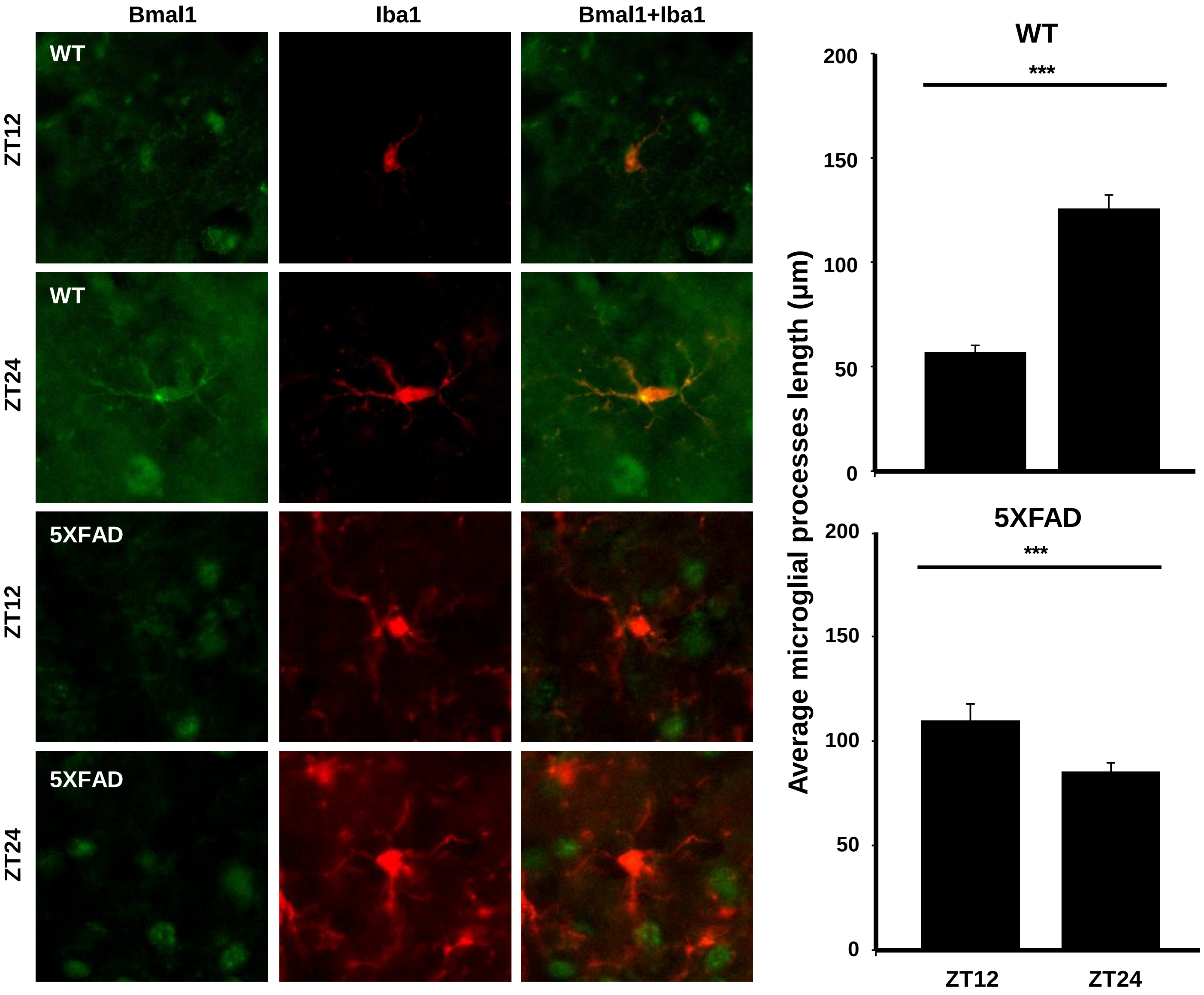

Figure S2. Microglial process length was higher at ZT24 than ZT12 and was decreased in 5XFAD along with Bmal1 downregulation. Representative images (left) and quantification (right) of the process length of microglia in the brain section of 6.5 month WT and 5XFAD mice at ZT12 and ZT24. ***P < 0.001.
